# Clinical characteristics of asymmetric hearing loss in children from a large pediatric cohort

**DOI:** 10.1038/s43856-026-01923-w

**Published:** 2026-09-25

**Authors:** Laura E. Hahn, Mario Fleischer, Friederike Wohlfarth, Jonas Althaus, Dirk Mürbe, Claudia Männel, Anke Hirschfelder

**Affiliations:** 1 Department of Audiology and Phoniatrics, Charité – Universitätsmedizin Berlin, Corporate Member of Freie Universität Berlin and Humboldt-Universität zu Berlin, Berlin, Germany; 2 Clinic of Otorhinolaryngology-Head and Neck Surgery, University Hospital Zurich, Zurich, Switzerland; 3 Max Planck Institute for Human Cognitive and Brain Sciences, Leipzig, Germany

**Keywords:** Paediatric research, Epidemiology

## Abstract

**Background:**

Asymmetric hearing loss (HL) is a common pediatric condition, characterized by differences in HL severity between both ears. Anamnestic and audiologic profiles of affected children are sparse, hindering development of evidence-based clinical guidelines.

**Methods:**

Clinical characteristics for children with bi- and unilateral asymmetric HL were retrospectively obtained from a register of children with permanent HL in Germany. Cohorts were stratified by laterality (uni-/bilateral) and presence of comorbidities (with/without). Between-group differences were assessed using absolute standardized mean differences.

**Results:**

Here we show, across uni- and bilateral groups, family history of HL is reported for approximately 25% and comorbidities for 40%-50% of children. Children with bilateral HL have a higher proportion of pre-/perinatal risk factors (e.g., hypoxia, artificial ventilation) than children with unilateral HL. Hereditary HL is more common in children with bilateral HL, whereas unexplained causes of HL is more frequent in unilateral cases. Sensorineural HL is predominant across groups; conductive or combined HL occurs more often in children with unilateral HL and comorbidities. Sloping and flat audiogram configurations are common across groups, though many children also exhibit other shapes. Notably, 60% of children with bilateral HL present different audiogram configurations in their left and right ears.

**Conclusion:**

Children with asymmetric HL represent a heterogeneous population with diverse clinical characteristics. This study highlights the need for comprehensive audiologic examination and tailored technical intervention. Bilateral asymmetries may pose greater clinical challenges compared to unilateral cases, due to higher incidence of comorbidities and risk factors as well as the high incidence of inter-aural differences in audiogram configurations.

## Plain language summary

Children with impaired hearing often show more severe hearing loss in one ear, known as asymmetric hearing loss. This study analyzed data from a medical register in Germany to examine clinical characteristics of children with hearing loss in one ear (and a healthy other ear) and children with hearing loss in both ears, but to different degrees. Both groups had similar rates of family history of hearing loss and comorbidities, but birth-related risk factors were more common in children with both ears impaired. Hearing-loss shapes varied widely and often differed between ears of the same child. These findings highlight the complexity of asymmetric hearing loss and can be used to establish more tailored clinical guidelines and technical interventions for children that are affected by asymmetric hearing.

## Abstract (web summary)

Hahn et al., analysed clinical and audiological characteristics of children with unilateral or bilateral asymmetric hearing loss from a large German registry, stratifying cases by laterality and comorbidity status. Children with bilateral asymmetric hearing loss show more hereditary causes and pre/perinatal risk factors, whereas unilateral cases more often have unexplained causes; sensorineural loss predominates, and 60% of bilateral cases have different audiogram configurations between ears.

## Introduction

Pediatric hearing loss (HL) is a common sensory deficit, affecting around 70 million children worldwide^1^. Hearing loss in children can be more severe in one ear than the other (i.e., asymmetric). In asymmetric bilateral HL (AHL), both ears show HL to some degree, but the severity is higher in one ear than the other. In unilateral HL (UHL), only one ear is affected while the second ear has normal hearing (NH). In patients with asymmetric HL, higher rather than lower frequencies are affected, and inter-aural differences in hearing-loss severity may be more pronounced in children than in adults^2^.

Currently, there is no consensus as to which audiometric configuration qualifies as “asymmetric”^3^, resulting in widely varying prevalence rates. For example, asymmetric HL has been reported to affect between 2.77% and up to 25.05% of the adult US-American population^4^ and up to 50% of adult patients with HL^5^, depending on the exact audiometric criterion. For children, prevalence rates for AHL as opposed to symmetric bilateral HL have not been explicitly reported so far. Yet, UHL affects between 3 and 6% of US-American school children^6^ and progresses into bilateral HL in at least one in ten children^7^. A recent meta-analysis indicates 8.9% of children are affected by bilateral or unilateral sensorineural HL at the speech frequencies from 15 dB HL onwards^8^. In a sample of 218 Finnish children with HL, the ratio of asymmetric to symmetric HL was around 1:2, with asymmetric HL being classified as an inter-aural difference of more than 10 dB HL in at least two frequencies^9^. These numbers indicate asymmetric HL to be a common pediatric condition, yet reports on clinical characteristics of affected children are sparse. Consequently, consensus groups call for large-scale studies with explicitly defined cohorts with asymmetric HL to better estimate, for example, risk factor prevalence^10^.

Asymmetric HL in pediatric patients poses complex demands on diagnostics and treatment, but current medical guidelines on pediatric HL cover asymmetric HL only scarcely (but see ref. ^11^). For one, bilateral asymmetries with substantial inter-aural threshold differences may warrant bimodal fitting, posing challenges to rehabilitation^12^. In those cases, two devices need to be fit, often by different clinicians at different institutions, as cochlear implants (CIs) need to be fitted in specialized pediatric CI-centers, whereas behind-the-ear hearing aids (BTE-HAs) are typically fitted by pediatric acousticians. Bimodal fitting also requires careful assessment of the individual contribution of each device as well as their combined interaction, for example, concerning loudness scaling, with only limited feedback available from young (preverbal) patients. Second, children with asymmetric HL need to be frequently monitored, given the high prevalence of hearing deterioration in the worse as well as the better-hearing ear^13,14^. Finally, inner-ear anomalies such as cochlear dysplasia or enlarged vestibular aqueducts, warrant extensive MRI- or CT-screenings^15^. In pediatric patients, especially in infants and pre-school children, however, imaging can be challenging due to discomfort, limited cooperation, the possible need for sedation, additional appointments, and restricted scanner availability, all of which may delay diagnosis and treatment.

Delayed or inadequate treatments of HL asymmetries cause long-term limitations of binaural hearing, resulting in cortical over-representation of the better-hearing ear (i.e., “aural preference syndrome”^16^), with far-reaching cascading effects on language, memory, and attention^17,18^. Children with UHL, in particular, may initially show normal language development and unimpaired speech perception in quiet listening conditions. Yet later during development, constant constraints on spatial and binaural hearing contribute to difficulties in higher-level cognition, such as speech-perception in noise, working-memory, and executive functioning^19,20^, placing these children at higher risk for educational and psycho-social problems^21,22^.

The current study characterizes risk factors, comorbidities, and associated findings, as well as audiologic characteristics in a large pediatric cohort with asymmetric bilateral HL (AHL) and unilateral HL (UHL). The study sample was derived from the German Registry for Hearing Loss in Children^23,24^, a medical registry holding data of children with permanent HL from more than 200 cooperating clinics and ped-audiologic practices in Germany. Risk factors, comorbidities, and associated findings were more common in children with AHL than UHL. Both groups present with heterogeneous audiogram configurations, often differing between the two ears. The presented findings allow for evidence-based decision-making in this understudied pediatric condition that poses complex demands on diagnostics and treatment.

## Methods

### Patient recruitment and grouping

The study was approved by the Ethical Board of the Medical Faculty of Charité – Universitätsmedizin, Berlin (EA2/002/24). Informed consent was obtained from legal guardians, and the study has been performed in accordance with the Declaration of Helsinki. Data for the current study were obtained in July 2024 from the German Registry for Hearing Loss in Children^23,24^. The registry includes 16,425 pediatric patients with permanent hearing loss of ≥20 dB HL in at least one ear, regardless of etiology or laterality, reported voluntarily by participating ENT-specialists and pediatric audiologists. Children were registered between 1994 and 2024. Audiometric data from at least one ear were available for 16,229 children. From the registry, children with AHL (*n* = 783, 4.77% of the entire registry) were identified based on the following criteria: at least 20 dB HL in each ear and an inter-aural difference of at least 30 dB HL at two or more frequencies (0.5, 1, 2, and 4 kHz), to rule out differences possibly originating from test-retest variance. Children with UHL (*n* = 2412, 14.69% of the entire registry) were selected based on the following criteria: a unilateral hearing loss of at least 35 dB HL (averaged across 0.5, 1, 2, and 4 kHz; thus excluding children with mild UHL^25^) and normal hearing at the other ear (<20 dB HL)^26^. For each child, the registry contains (1) anamnestic information on family history, risk factors, and comorbidities, and (2) audiologic information such as HL type, laterality, progression, and mean threshold; as provided by the child’s ENT-specialist or pediatric audiologist, and were ascertained upon inclusion in the registry. Owing to the general delay between diagnosis and registry enrollment, the reporting physician was typically able to assess and confirm hearing-loss progression over time. Until 2014, information on hearing-loss progression was additionally verified by the study team through personal communication with each child’s physician. After 2014, such direct verification was impeded by changes in General Data Protection Regulation (GDPR) restrictions. Hearing-loss progression was categorized by the treating physician as “secured” (documented audiometric deterioration over time), “likely” (clinical suspicion of deterioration without definitive serial confirmation), “no progression” (stable thresholds), or “unspecified” (insufficient information to determine progression despite clinical evaluation). Information concerning comorbidities or associated findings was registered as free-text strings within four variables: “underlying impairment” (e.g., neuroblastoma), “additional impairments” (e.g., epilepsy), “craniofacial malformations” (e.g., microtia), and “syndrome” (e.g., Down syndrome), reflecting the original labels for the registry reporting sheet. No formal etiological distinction was imposed by the investigators, and all free-text entries were recorded as documented and subsequently dichotomized into the presence versus absence of any comorbidity or underlying associated findings. Family history of HL was coded as “yes”, when parents, siblings and/or other relatives suffered from HL. Risk factors for each child were indicated in a list of twelve possible risk factors, typically associated with pediatric HL, with zero to multiple responses per child. Audiometric thresholds (audiograms) were available for *n* = 3978 ears in total from four frequencies (0.5, 1, 2, and 4 kHz) and were derived from auditory brainstem responses (*n* = 1261 children, median age of diagnosis: 0.64 years, IQR: 0.31–2.85) or pure-tone air-conduction audiometry (*n* = 1886 children, median age of diagnosis: 5.84 years, IQR: 4.52–7.56). For *n* = 48 children, the audiometric method was unclear. Depending on the audiologic method, the average HL was calculated as the arithmetic mean of the available frequency-specific thresholds across 0.5, 1, 2, and 4 kHz. For auditory brainstem response, the calculation was based on at least one AEP-threshold, whereas for pure-tone audiometry, the mean was derived from at least three thresholds.

### Statistics and reproducibility

All data were analyzed in R, using R studio^25^, see Supplementary Materials for all data and analysis scripts. Anamnestic and audiologic variables were summarized as proportions, excluding missing values, for the AHL and UHL groups using the gtsummary package^27^ and compared based on the absolute standardized mean differences (ASMDs) using a custom-made function. An ASMD above 0.10 represents an imbalance (i.e., difference) between groups, and a larger ASMD suggests a larger between-group difference for the respective variable^28,29^. Proportions for risk factors and audiologic variables were stratified into four subgroups according to the type of HL (AHL, UHL) and presence of comorbidities (with, without). Audiograms were classified into one of five possible patterns^2^: *sloping, rising, flat, U-shaped* and *tent-shaped*, based on the sum of their shape similarity (https://pypi.org/project/shapesimilarity/) and R-squared. For each frequency, values ranged between 0 and 120 dB HL, thus excluding thresholds indicating no response at the limit of most audiometers. Individual audiograms per ear were averaged per subgroup (AHL vs. UHL, with vs. without comorbidities) and per grade of HL (grades according to Ref. [^26^]). Given the large number of possible comparisons between audiogram proportions, only subgroup differences of at least 10% are reported for this variable.

## Results

### Anamnestic information

Demographic etiologic variables are summarized in Table 1 for *n* = 783 children with AHL and *n* = 2412 children with UHL, 53% *males* in each group.

**Table 1:** Demographic and etiologic information of patient groups with AHL and UHL

Characteristics	*N*	AHL^a^	*N*	UHL^a^	ASMD^b^	
Entire sample						
Sex	782		2412			
Female		365 (47%)		1130 (47%)	0.00	
Male		417 (53%)		1282 (53%)	0.00	
Missing		1		0		
Hearing loss origin	777		2393			
Acquired		132 (17%)		320 (13%)	0.10	
Hereditary		186 (24%)		390 (16%)	0.20	
Unexplained		459 (59%)		1683 (70%)	**0.24**	
Missing		6		19		
Number of risk factors	783		2412			
0		502 (64%)		1844 (76%)	**0.28**	
1		181 (23%)		388 (16%)	0.18	
2		56 (7.2%)		99 (4.1%)	0.14	
3 or more		44 (5.6%)		81 (3.4%)	0.12	
Comorbidities or associated findings	783		2412			
With		392 (50%)		1067 (44%)	0.12	
Without		365 (47%)		1248 (52%)	0.10	
Not specified		26 (3.3%)		97 (4.0%)	0.04	
With comorbidities or associated findings	392		1067			
Additional impairment(s)		210 (54%)		413 (39%)	**0.30**	
Underlying impairment(s)		229 (58%)		603 (57%)	0.04	
Syndrome(s)		108 (28%)		145 (14%)	**0.35**	
Craniofacial malformation(s)		156 (40%)		480 (45%)	0.11	
Family history of hearing loss	783		2412			
With		196 (25%)		515 (21%)	0.09	
Without		430 (55%)		1417 (59%)	0.08	
Not specified		157 (20%)		480 (20%)	0.00	
With a family history of hearing loss	196		515			
Parent(s)		75 (38%)		164 (32%)	0.14	
Sibling(s)		54 (28%)		66 (13%)	**0.40**	
Other relative(s)		125 (64%)		377 (73%)	**0.21**	

Note that for family history and comorbidities, multiple responses per child were possible.

^a^*n* (%).

^b^ASMD = absolute standardized mean difference AHL vs. UHL, ASMDs >0.2 highlighted in bold.

*Age of diagnosis* was mainly within the preschool years for both cohorts. Median age of diagnosis was 3.26 years (IQR 0.73–5.62) for children with AHL and 4.90 years (IQR 0.70–6.57) for children with UHL.

*Hearing-loss origin* was mostly unexplained, with a larger proportion of children with UHL than AHL (59% AHL vs. 70% UHL, ASMD: 0.24). Proportions for *acquired* and *hereditary* HL were slightly larger in children with AHL than UHL (*acquired HL*: 17% AHL vs. 13% UHL, ASMD: 0.10; *hereditary HL*: 24% AHL vs. 16% UHL, ASMD: 0.20). Note that “unexplained” refers to cases in which no etiological cause for the HL could be identified despite clinical evaluation, and should not be confused with cases in which no information on HL origin was provided, denoted as “missing” in Table 1.

*Risk factors* were indicated for more than *n* = 800 children in total (Table 1), representing 36% of children with AHL and 23% with UHL. The group of children free of any risk factors was larger in children with UHL than AHL (64% AHL vs. 76% UHL, ASMD: 0.28). Once a risk factor was present, most children were affected by a single risk factor, as opposed to two or more risk factors, with only small group differences (Table 1). Occurrence rates for all possible risk factors are listed in Table 2, stratified by subgroup. Across all four subgroups, risk factors associated with the *pre- and perinatal period* (e.g., *pre-term birth, low birth weight, hypoxia, and artificial ventilation*) were more common than most other risk factors and were particularly frequent in children with AHL suffering from comorbidities (i.e., >4% for most pre- and perinatal risk factors). In addition, 8.9% of the children with AHL were affected by *meningitis* and 4.8% with UHL in the subgroups with comorbidities (compared to <1% in the subgroups without comorbidities). Children with UHL and comorbidities were also frequently affected by *pre- and post-natal infections* (7%). For children with AHL, *consanguineous parents* were another frequent risk factor (5.8 to 8.8% in both subgroups). *Ototoxic medication* was reported for 2.7 to 4.6% of children with comorbidities and 1.0 to 1.9% without comorbidities. Across subgroups with and without comorbidities, children with UHL had lower proportions than children with AHL for most risk factors. Group differences between children with AHL and UHL affected by comorbidities were largest for *artificial ventilation* (12% AHL vs. 4.9% UHL, ASMD: 0.29) and *hypoxia* (10% AHL vs. 4.4% UHL, ASMD: 0.26), each affecting more children with AHL than UHL. In children without comorbidities, the largest group differences were present for *consanguineous parents*, affecting more children with AHL than UHL without comorbidities (8.8% AHL vs. 3.5% UHL, ASMD: 0.25). Note that *unspecified risk factors* were common in all subgroups (8.1–16.0% of children, Table 2).

**Table 2:** Occurrence rates of risk factors within subgroups (AHL vs. UHL, with vs. without comorbidities or associated findings)

	With comorbidity			Without comorbidity		
Risk factor	AHL, *N* = 392^a^	UHL, *N* = 1067^a^	ASMD﻿^b^	AHL, *N* = 365^a^	UHL, *N* = 1248^a^	ASMD^b^
5-Min Apgar score ≤3	15 (3.8%)	13 (1.2%)	0.19	1 (0.3%)	5 (0.4%)	0.02
Artificial ventilation	**47 (12%)**	52 (4.9%)	**0.29**	14 (3.8%)	30 (2.4%)	0.09
Consanguinity	**20 (5.8%)**	35 (3.5%)	0.11	**30 (8.8%)**	41 (3.5%)	**0.25**
Head trauma with inpatient treatment	5 (1.3%)	22 (2.1%)	0.06	1 (0.3%)	5 (0.4%)	0.02
Hyperbilirubinemia with exchange transfusion	4 (1.0%)	8 (0.7%)	0.03	0 (0%)	5 (0.4%)	0.07
Hypoxia	**41 (10%)**	47 (4.4%)	**0.26**	**20 (5.5%)**	31 (2.5%)	0.17
Low birthweight (<1500 g)	18 (4.6%)	25 (2.3%)	0.13	5 (1.4%)	18 (1.4%)	0.01
Maternal substance abuse	6 (1.5%)	6 (0.6%)	0.11	1 (0.3%)	7 (0.6%)	0.04
Meningitis/encephalitis	**35 (8.9%)**	51 (4.8%)	0.18	2 (0.5%)	0 (0%)	0.16
Ototoxic medication	18 (4.6%)	29 (2.7%)	0.11	7 (1.9%)	13 (1.0%)	0.07
Pre- and post-natal infections	**25 (6.4%)**	**75 (7.0%)**	0.03	10 (2.7%)	25 (2.0%)	0.04
Premature birth (≤32 GW)	**33 (8.4%)**	47 (4.4%)	0.18	9 (2.5%)	42 (3.4%)	0.05
Unspecified risk factor	**64 (16%)**	**142 (13%)**	0.09	**39 (11%)**	**101 (8.1%)**	0.09

*GW* gestational week.

^a^*n* (%).

^b^*ASMD* absolute standardized mean difference AHL vs. UHL, ASMDs >0.2 highlighted in bold. Risk factors with proportions ≥5% highlighted in bold. Note that children could be affected by multiple risk factors.

*Comorbidities or associated findings* alongside the HL were indicated for around 50% of children in both groups, but with a slightly larger proportion for children with AHL than UHL (50% AHL vs. 44% UHL, ASMD: 0.12). Among children with comorbidities, more than half of the children with AHL had *underlying impairments* (58%) and/or *additional impairments* (54%). For children with UHL, *underlying impairments* were most common (57%). Moderate differences between groups were observed in the number of children affected *by additional impairments* (54% AHL vs. 39% UHL, ASMD: 0.30) and *syndromes* (28% vs. 14%, ASMD: 0.35), each with more children with AHL being affected than children with UHL.

*Family history* of HL was indicated for about 25% of children in both groups. Among children with a family history, *other relatives* (not parents or siblings) were most frequently affected in both groups, but with a larger proportion for children with UHL than AHL (64% AHL vs. 73% UHL, ASMD: 0.21). *Siblings* were affected more often in children with AHL than UHL (28% AHL vs. 13% UHL, ASMD: 0.40).

### Audiologic information

Audiologic information is given in Table 3 for the four subgroups (stratified by AHL vs. UHL and with vs. without comorbidities).

**Table 3:** Audiologic information of children with AHL and UHL stratified by the presence of comorbidities or associated findings

	With comorbidity					Without comorbidity				
Characteristics	*N*	AHL^a^	*N*	UHL^a^	ASMD^b^	*N*	AHL^a^	*N*	UHL^a^	ASMD^b^
Hearing loss type	702		1042			676		1215		
Combined		115 (16%)		88 (8.4%)	**0.24**		34 (5.0%)		103 (8.5%)	0.14
Conductive		57 (8.1%)		353 (34%)	**0.66**		9 (1.3%)		32 (2.6%)	0.09
Sensorineural		530 (75%)		601 (58%)	**0.39**		633 (94%)		1080 (89%)	0.17
Progression	392		1067			365		1248		
Secured		29 (7.4%)		47 (4.4%)	0.13		20 (5.5%)		52 (4.2%)	0.06
Likely		41 (10%)		66 (6.2%)	0.16		36 (9.9%)		62 (5.0%)	0.21
No		98 (25%)		437 (41%)	**0.33**		93 (25%)		292 (23%)	0.05
Unspecified		224 (57%)		517 (48%)	0.17		216 (59%)		842 (67%)	0.17
Worse-ear threshold (dB HL)	310	81 (20)	947	66 (21)	–	278	82 (22)	1111	68 (23)	–
Better-ear threshold (dB HL)	310	41 (19)		–	–	278	42 (20)		–	–
Inter-aural difference (dB HL)	310	40 (16)		–	–	278	39 (17)		–	–
Worse-ear side	392		1067			365		1248		
Left		201 (51%)		530 (50%)	0.03		186 (51%)		651 (52%)	0.02
Right		191 (49%)		537 (50%)	0.03		179 (49%)		597 (48%)	0.02
Worse-ear HL-grade	392		1067			365		1248		
Moderate		8 (2.0%)		219 (21%)	**0.52**		15 (4.1%)		288 (23%)	**0.50**
Moderately severe		69 (18%)		302 (28%)	**0.25**		58 (16%)		271 (22%)	0.14
Severe		76 (19%)		199 (19%)	0.02		58 (16%)		193 (15%)	0.01
Profound		69 (18%)		102 (9.6%)	**0.25**		51 (14%)		161 (13%)	0.03
Complete or total		170 (43%)		245 (23%)	**0.46**		183 (50%)		335 (27%)	**0.51**
Worse-ear audiogram	231		536			203		773		
Flat		94 (41%)		197 (37%)	0.08		60 (30%)		236 (31%)	0.02
Rising		18 (7.8%)		65 (12%)	0.14		11 (5.4%)		77 (10.0%)	0.16
Sloping		62 (27%)		130 (24%)	0.06		75 (37%)		266 (34%)	0.05
Tent-shaped		24 (10%)		80 (15%)	0.13		15 (7.4%)		67 (8.7%)	0.05
U-shaped		33 (14%)		64 (12%)	0.07		42 (21%)		127 (16%)	0.11
Better-ear audiogram	332					288				
Flat		118 (36%)		–			90 (31%)		–	
Rising		55 (17%)		–			31 (11%)		–	
Sloping		72 (22%)		–			83 (29%)		–	
Tent-shaped		44 (13%)		–			38 (13%)		–	
U-shaped		43 (13%)		–			46 (16%)		–	
Mismatch audiogram	213					190				
Match		83 (39%)		–			82 (43%)		–	
Mismatch		130 (61%)		–			108 (57%)		–	

Ns represent children; only for “Hearing-loss type,” Ns refer to ears. Worse/better ear dB HL values were limited to a maximum of 120 dB HL; HL grades are according to ref. ^26^.

^a^*n* (%); Mean (SD).

^b^ASMD = absolute standardized mean difference AHL vs. UHL, ASMDs >0.2 highlighted in bold.

*Hearing loss type* was mostly sensorineural in all subgroups. Pronounced differences in proportions for HL types were observed in the groups of children with comorbidities. While children with AHL and comorbidities were more likely to have *sensorineural* (75% AHL vs. 57% UHL, ASMD: 0.39) or *combined HL* (16% AHL vs. 8.4% UHL, ASMD: 0.24), children with UHL and comorbidities more often had *conductive HL* (8.1% AHL vs. 34% UHL, ASMD: 0.66). Note that HL etiology of *conductive HL* in children with UHL was mostly *hereditary* (36%) and rarely *acquired* (10%).

*Progression of hearing loss* was indicated as *unspecified* for most children (>47% in all subgroups), and few children had a *secure progression* (<7.4% in all subgroups).

The average *threshold* of children with AHL (worse-ear thresholds) was 91 dB HL (range: 41 to >120 dB HL) in children with comorbidities and 93 dB HL (range: 36 to >120 dB HL) in children without comorbidities. Children with UHL and comorbidities had an average of 73 dB HL (range: 35 to >120 dB HL) and 75 dB HL (range: 35 to >120 dB HL) when no comorbidities were present. The *average inter-aural threshold difference* was 47 dB HL in children with AHL with comorbidities (range: 15–110 dB HL) and 42 dB HL in children with AHL without comorbidities (range: 15–110 dB HL). *Left and right ears* were affected equally often in all subgroups.

*Degree of hearing loss* in the worst ears in children with AHL was mostly *complete/total* (>95 dB HL), regardless of whether they were affected by comorbidities (>40% in both AHL subgroups). *Moderate HL* (35–49 dB HL) occurred only in a few children in the subgroups with AHL (<5%). In children with UHL, proportions for HL degrees were more evenly distributed, regardless of whether comorbidities were present or not (proportions for all degrees from 9.6 to 28%). Consequently, *moderate HL* was much more common in children with UHL than AHL (ASMDs: ≥0.5), and *complete/total HL* occurred more often in children with AHL than UHL (ASMDs: >0.4).

**Fig. 1:**
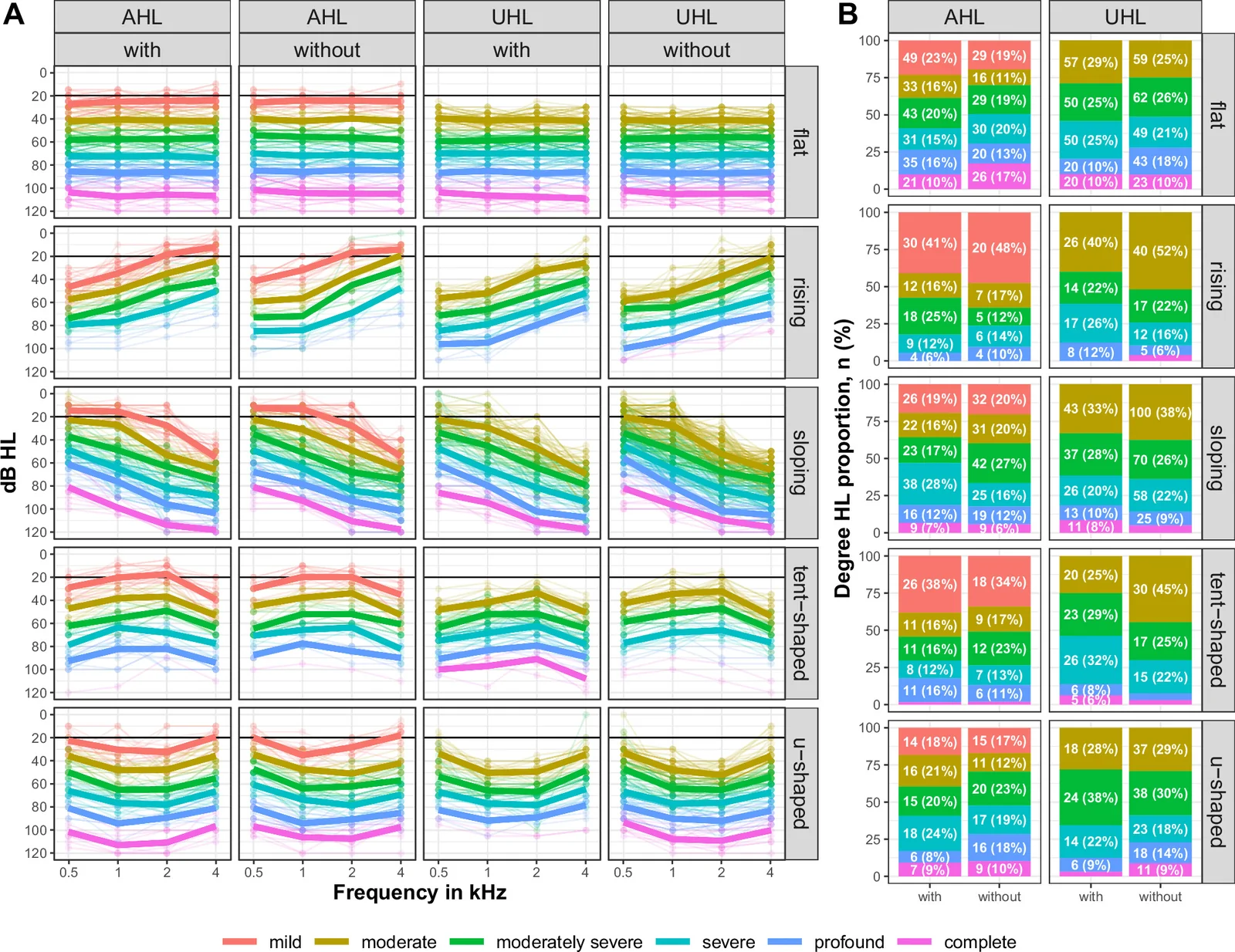
Audiograms for children with AHL and UHL stratified by the presence of comorbidities or associated findings (with vs. without). **A** Individual thresholds for *n* = 2363 ears, colored thick lines = averaged thresholds per HL-grade, black solid line = 20 dB HL, averages (thick lines) removed when *n* < 5. **B** Proportions of HL-grades^26^ per subgroup and audiogram configuration, values = *n* (%), labels removed when proportions are <6%.

*Audiogram configurations* (flat, rising, sloping, U-shaped, tent-shaped) are summarized in Table 3. Audiogram proportions adhered to commonly observed clinical patterns. Audiograms of worse ears from children with conductive HL predominantly showed flat, rising, and tent-shaped configurations (18–44%), while children with sensorineural HL predominantly had sloping configurations on their worse ear (36%). Across AHL and UHL subgroups and regardless of whether comorbidities were present, *flat* or *sloping* (high-frequency) were the most prevalent audiogram configurations, while around 30% of audiograms in all subgroups comprised *rising*, *tent-* or *U-shaped* configurations. Among children with AHL, around 60% had different audiogram configurations (*flat, rising, sloping, tent-* or *U-shaped*) on the left and right ear (see *Mismatching audiogram*, Table 3). Individual and average audiograms per subgroup (AHL vs. UHL, with vs. without comorbidities), degree of HL, and audiogram configuration (*flat, rising, sloping, tent-shaped, U-shaped*) are depicted in Fig. 1A, together with their corresponding proportions (Fig. 1B). Proportions for audiogram configurations were similar across subgroups and degree of HL (differences rarely >10%), and the five audiogram configurations were present within all subgroups, regardless of degree of HL. The only exception was *rising* and *tent-shaped* audiograms, which were scarce or absent in children with AHL and UHL with profound and complete HL. In the following, only the most prevalent degree of HL will be described per audiogram configuration, and AHL/UHL group (for further details, see Fig. 1B). For *flat audiograms*, no degree of HL was particularly prevalent in any of the subgroups. For *rising audiograms*, mild and moderate HL were the most prevalent degrees of HL in the AHL and UHL groups. Regarding *sloping audiograms*, the majority of children with UHL had moderate to moderately severe HL, while no degree of HL was particularly common in children with AHL. For *tent-shaped audiograms*, mild HL was the most common degree of HL for children with AHL. For children with UHL, moderate HL was prevalent in children without comorbidities, while there was no particularly common degree of HL for children with UHL and comorbidities. Regarding *U-shaped audiograms*, degrees of HL were mixed for children with AHL, while children with UHL mainly showed moderate and moderately severe degrees of HL.

## Discussion

The current study provided estimates for anamnestic and audiologic characteristics from a retrospective cohort of children with asymmetric hearing loss. We will first discuss results for anamnestic variables. Unexplained HL origin was more common in children with UHL than AHL, driven by the larger proportion of children with hereditary HL in children with AHL. A family history of HL was indicated for around 25% of children in both groups. For those affected by family history, siblings were affected more often in children with AHL than UHL, while other relatives were affected more often in children with UHL. Comorbidities or associated findings were indicated for 50% of children with AHL and for 44% with UHL (Table 1). Additional impairments, syndromes, and risk factors occurred more frequently in children with AHL than UHL. Common risk factors were related to the pre- and perinatal period (e.g., hypoxia, artificial ventilation) and pre- and post-natal infections, especially in children with AHL with comorbidities. Unspecified risk factors were also common. Between-group differences in risk-factor proportions were small and concerned hypoxia, artificial ventilation, and consanguineous parents, which were more common in children with AHL than UHL.

Henceforth, audiologic variables will be discussed. Sensorineural HL was prevalent in all groups. In children with comorbidities, combined and conductive HL were more frequent in children with UHL than AHL. HL progression was likely or secured for 9 to 15% of children in all groups. The grade of HL in worse ears in children with AHL was most often complete/total, while more variation in HL-grades was present in children with UHL. Audiograms of worse ears were typically *flat* or *sloping* (24–41% in all groups), and 60% of children with AHL had different audiogram configurations at their worse and better ears (Table 3). Proportions for audiogram configurations were similar across subgroups and HL-grades (differences rarely >10%). The following sections will highlight the most important findings and discuss some less expected observations.

Comorbidities and associated findings affected around half of the children with AHL and UHL, which is a higher proportion than the often-cited 20–40% of children with pediatric HL^30^, but in accordance with more recent studies^9,31^. Differences in the proportion of *types of comorbidities* within our sample reflect the heterogeneous etiology of asymmetric HL^21^. Importantly, children with UHL and comorbidities had a three-fold increase in proportion for conductive HL compared to the other subgroups (Table 3). While craniofacial malformations were equally frequent in UHL and AHL (20% in both groups), the registry does not systematically distinguish between specific subtypes, such as external ear canal atresia versus inner ear malformations. External ear anomalies, particularly ear canal atresia, are frequently unilateral and typically result in conductive HL, which may partly explain the higher proportion of conductive HL observed in UHL. Taken together, comorbidities in children with asymmetric HL seem to be equally prevalent as in the entire population with pediatric HL.

The proportion of children affected by at least one risk factor was higher for children with AHL than UHL, and risk factors were mainly reported for children with AHL and comorbidities (see Table 2). *Admission to the neonatal intensive care unit (NICU)*, a risk factor listed by the Joint Committee on Infant Hearing^32^, was not explicitly captured in our dataset, yet perinatal risk factors that likely result in *NICU admission* (*premature birth, syndromes, hypoxia, artificial ventilation, low birthweight, low Apgar score*) each affected between 3.8 to 28% of children with AHL and comorbidities, while being less frequent in the other subgroups (Tables 1 and 2). *Meningitis* has previously been identified as a major etiology of asymmetric HL^21,33^. Together with other post- and prenatal infections, meningitis was mainly reported for children with comorbidities, while being rare in children without comorbidities, and differences between AHL and UHL groups were negligible (Table 2). *Family history of HL*, another risk factor listed by the Joint Committee on Infant Hearing^32^, affected more children with AHL than UHL without comorbidities, while proportions were nearly equal in the subgroups with comorbidities. In conclusion, common risk factors of pediatric HL^32^ were also identified in the current sample of children with asymmetric HL. Children with AHL were more often affected by risk factors than children with UHL, and differences in risk factor proportions were largest for family history, consanguinity, hypoxia, and artificial ventilation.

*Consanguinity* is a well-known etiology of HL^25^, and previous studies suggested consanguinity to be associated with genetic non-syndromic HL in particular^34–36^. In our sample, consanguineous parents were indicated for 3.5 to 8.8% of children across subgroups, with higher rates for children with AHL than UHL and the highest values in children with AHL without reported comorbidities (8.8%, see Table 2). Children with AHL also had higher rates of hereditary HL in our sample. Levi al. reported consanguineous parents for 7% of children in a cohort of Jewish children with HL living in Jerusalem^36^. Our current study indicates similar rates in a cohort with asymmetric HL in central Europe. Interestingly, within our registry, the proportion of reported consanguineous parents has remained stable across the last 30 years (i.e., 7% in 2001^37^). This observation is unexpected, given that over the last decades, Germany has seen a rise in immigration^38^, including from regions where consanguineous marriages are common. As of now, consanguinity is not listed as a JCIH risk factor^32^. In light of the current findings, however, we agree with other initiatives^39^ that advocate for advanced hearing screening of children from consanguineous parents. Additionally, future studies should differentiate between risk factors specifically associated with sensorineural HL and risk factors more common in children with permanent conductive HL.

Within the sample with UHL, 33% of children had a unilateral sensorineural HL of at least 71 dB HL or more (*n* = 789 children, 48% females), thus presenting with single-sided deafness (SSD)^40^. Only 2% of these SSD patients were registered with cochlear malformations and nerve deficiencies (*n* = 13), such as Mondini dysplasia, aperture stenosis, or agenesia, suggesting that the majority of SSD patients within our sample might qualify as CI candidates^41,42^. Previous studies reported much higher rates for cochlear-nerve deficiencies within pediatric SSD samples (25% in ref. ^41^; 44% in ref. ^43^). The small percentage in our study presumably results from the nature of the current registry: Records date back as far as 1994, when MRI and CT scans were less common for children with HL. In Germany, CI treatment for pediatric SSD patients has only been approved in 2013^44^. Radiologic examination of SSD children before this time was thus mainly driven by etiologic, rather than therapeutic interest.

For 60% of children with AHL, different audiogram configurations at their worst and better ears were recorded (*mismatching audiograms*, Table 3). Epidemiological studies on audiometric configurations in children with asymmetric HL are scarce. Here, we show that for 60% of children with bilateral asymmetric HL, their two ears do not only differ in HL severity, but also in their audiogram configuration. These inter-aural differences might contribute to heterogeneity in outcomes of children with AHL and pose complex demands on audiometric examination and technical intervention^45^. Specifically, audiograms allow for better determination of which listening situations are particularly challenging for an individual. For example, patients with sloping audiograms, which affect merely high frequencies, suffer most in noisy situations, while quiet surroundings often pose little challenge. Therefore, any two children with the same degree of HL might face different challenges in their everyday lives, depending on their individual audiogram configurations. Information on audiogram configuration can thus extend the standard classification of HL severity by a valuable factor that allows generalization towards children’s real-world listening experience^46^.

*Flat* or *sloping* audiograms were common in our sample, regardless of whether comorbidities were present (Table 3). While the observed dominance of flat and sloping audiograms is in accordance with previous studies^9,47^, audiograms in our study were also diverse: Alongside the dominant *flat* and *sloping* configurations, *rising, U- and tent-shaped* configurations frequently occurred at worse and better ears in all subgroups irrespective of the individual HL-grade (Fig. 1). Moreover, *rising* audiograms were observed more often for children with UHL (Table 3). More heterogenous audiogram configurations for children compared to adults have been reported before, with adults mainly showing *sloping* and *U-shaped* configurations^2^. With the current data, we extend this notion to children affected by asymmetric HL. Future studies should relate audiogram configurations to future outcome measures, such as speech or speech-in-noise comprehension. This would allow us to move beyond descriptive categorization towards predictive models of communicative performance.

Data for this cohort were provided from 1994 onwards by more than 200 participating institutions across Germany. As participation was not nationwide and data submission was only voluntary, the present results may not fully represent the entire German population. Additionally, since the registry was established already in the mid-1990s, the present study lacks information on more recent epidemiological and clinical developments, such as genetic screening, radiologic work-up, treatment outcomes, and longitudinal development of HL progression. The long observational time span of the registry may bias the observed proportions. In particular, the implementation of the universal newborn hearing screening (UNHS) in Germany in 2009 substantially improved early detection and diagnostic precision of pediatric hearing loss. Children registered before UNHS introduction were often diagnosed later, and information on hearing status in the first years of life was limited or incomplete^48^. This may have resulted in delayed classification or misclassification of hearing loss characteristics reported to the registry. For example, asymmetric HL recorded at later ages may have been congenital unilateral HL that was not detected at birth. Furthermore, anamnestic information provided for each child may be biased towards more apparent conditions and risk factors, such as syndromes and craniofacial malformations, resulting in under-reporting of more subtle defects. In addition, the registry does not document the diagnostic modality used to identify malformations (e.g., MR/CT imaging versus clinical examination alone). Therefore, the reported prevalence of malformations may reflect heterogeneous diagnostic practices and potential under-detection of radiologically identifiable anomalies. Determination of true incidence thus requires further, refined research. Nevertheless, our large sample size and broad scope of analyses provide a reliable overview of the clinical characteristics of children with asymmetric HL. Future studies should follow up on the estimates provided here with more focused prospective analyses.

## Conclusions

Children with permanent bi- and unilateral asymmetric HL represent a heterogeneous population regarding their clinical characteristics. Our findings highlight a need for audiologic work-up as well as technical intervention that is specifically targeted towards children with asymmetric HL. Children with bilateral AHL might pose a particularly challenging group of patients, due to the increased incidence of comorbidities and associated findings, as well as risk factors, compared to children with unilateral HL. Sloping and flat audiogram configurations were common in both children with bi- and unilateral asymmetric HL, but a large proportion of audiograms was accounted for by other configurations, again suggesting heterogeneity in clinical phenotypes. For 60% of children with AHL, audiogram configurations differed between their left and right ears, again posing complex demands on the clinical management of affected children.

## Data Availability

Raw data, including source data underlying Fig. 1 and all Tables 1–3, can be found at the following link: 10.5281/zenodo.20280593^49^. Access to the German Registry for Hearing Loss in Children is limited but may be provided upon reasonable request to the authors.

## Code availability

Analysis scripts and subsequently derived results are available at 10.5281/zenodo.20280593^49^.
